# Diet Supplementation in ω3 Polyunsaturated Fatty Acid Favors an Anti-Inflammatory Basal Environment in Mouse Adipose Tissue

**DOI:** 10.3390/nu11020438

**Published:** 2019-02-20

**Authors:** Cecilia Colson, Rayane A. Ghandour, Océane Dufies, Samah Rekima, Agnès Loubat, Patrick Munro, Laurent Boyer, Didier F. Pisani

**Affiliations:** 1 Université Côte d’Azur, CNRS, Inserm, iBV, 06107 Nice, France; Ceciclia.Colson@unice.fr (C.C.); ghandourrayane@hotmail.com (R.A.G.); Samah.Rekima@unice.fr (S.R.); Agnes.Loubat@unice.fr (A.L.); 2 Université Côte d’Azur, Inserm, C3M, 06107 Nice, France; oceane.dufies@unice.fr (O.D.); Patrick.Munro@unice.fr (P.M.); Laurent.Boyer@unice.fr (L.B.); 3 Didier Pisani, Laboratoire de PhysioMédecine Moléculaire—LP2M, Univ. Nice Sophia Antipolis, 28 Avenue de Valombrose, 06107 Nice CEDEX 2, France

**Keywords:** oxylipins, brown adipose tissue, white adipose tissue, macrophages, inflammation

## Abstract

Oxylipins are metabolized from dietary ω3 and ω6 polyunsaturated fatty acids and are involved in an inflammatory response. Adipose tissue inflammatory background is a key factor of metabolic disorders and it is accepted that dietary fatty acids, in terms of quality and quantity, modulate oxylipin synthesis in this tissue. Moreover, it has been reported that diet supplementation in ω3 polyunsaturated fatty acids resolves some inflammatory situations. Thus, it is crucial to assess the influence of dietary polyunsaturated fatty acids on oxylipin synthesis and their impact on adipose tissue inflammation. To this end, mice fed an ω6- or ω3-enriched standard diet (*ω6/ω3* ratio of 30 and 3.75, respectively) were analyzed for inflammatory phenotype and adipose tissue oxylipin content. Diet enrichment with an ω3 polyunsaturated fatty acid induced an increase in the oxylipins derived from ω6 linoleic acid, ω3 eicosapentaenoic, and ω3 docosahexaenoic acids in brown and white adipose tissues. Among these, the level of pro-resolving mediator intermediates, as well as anti-inflammatory metabolites, were augmented. Concomitantly, expressions of M2 macrophage markers were increased without affecting inflammatory cytokine contents. In vitro, these metabolites did not activate macrophages but participated in macrophage polarization by inflammatory stimuli. In conclusion, we demonstrated that an ω3-enriched diet, in non-obesogenic non-inflammatory conditions, induced synthesis of oxylipins which were involved in an anti-inflammatory response as well as enhancement of the M2 macrophage molecular signature, without affecting inflammatory cytokine secretion.

## 1. Introduction

ω6 linoleic acid (LA), a precursor of dihomo-γ-linolenic acid (DGLA) and arachidonic acid (ARA), and ω3 α-linolenic acid, a precursor of eicosapentaenoic (EPA) and docosahexaenoic (DHA) acids are essential polyunsaturated fatty acids (PUFAs) only supplied by food. These PUFAs are required for healthy development from embryonic steps to adult life and are involved in a variety of biological processes, especially, in adipose tissue [1,2]. It is now well accepted that insufficient intakes of ω3 PUFAs, as well as an excess of ω6 PUFAs, correlate with various diseases; especially, metabolic diseases [3,4,5]. For example, ARA intake correlates positively with being overweight/obese, inflammatory diseases, and associated metabolic syndrome [6,7,8,9,10]. Indeed, ω6 oxylipins (oxygenated derivatives of PUFAs) are known to favor inflammatory responses [11], as well as to promote energy storage [12] and to inhibit energy expenditure [13,14]. The dietary *ω6/ω3* PUFAs ratio is more important than the total amount of PUFA intake as it determines the level of synthesized ω6-derived oxylipins. Indeed, ω3 PUFAs modulate ω6-derived oxylipins synthesis [15]. Mechanistically this is characterized by (i) the capacity of ω6 and ω3 PUFAs to compete at the level of lipoxygenase (LOX) and cyclooxygenase (COX), their two major metabolization pathways and (ii) the capacity of various ω3 PUFAs to inhibit these pathways. 

The increase in the number of overweight or obese people has reached an epidemic stage in the 21st century. More than 2 billion adults are overweight (body mass index (BMI) > 25 kg/m^2^) and at least 600 million are clinically obese (BMI > 30 kg/m^2^). Obesity and being overweight are the consequences of a positive energy balance that leads to an increase in the mass of subcutaneous and visceral white adipose tissue. White adipocytes are storing energy under the form of triglycerides whereas brown adipocytes dissipate energy from triglycerides by producing heat (=thermogenesis). In addition, white and brown adipocytes are able to secrete molecules acting on their environment, and especially, on immune cells [16]. For example, white adipocytes secrete adipokines (e.g., adiponectin) and pro-inflammatory factors (e.g., PAI-1, MCP-1, or IL-6) which are able to recruit and activate macrophages [17]. Furthermore, it has also been shown that the white adipose tissue of obese subjects is characterized by low-grade inflammation that can lead to metabolic disorders such as insulin resistance [18]. This inflammation, characterized by an increase in inflammatory markers such as TNFα, PAI-1, or interleukins 1 and 6 (IL-1, IL-6), promotes the macrophage infiltration of adipose tissue and the polarization of macrophages of the alternative M2 type in classic pro-inflammatory M1 type [19]. 

The macrophages respond to environmental cues by acquiring specific functional phenotypes. Pro-inflammatory M1 macrophages are involved in the fight against many infections. They are activated by Toll-like receptor (TLR) ligands such as lipopolysaccharide and saturated fatty acids, but also by IFNγ and TNFα. They participate in the inflammatory environment by secreting many cytokines such as IL-1, IL-6, IL-12, IL-23, and TNFα, and by participating in the chemo-attraction of other immune cells [20]. M2 macrophages are more heterogeneous at functional and secretory levels. Considered as anti-inflammatory or inactive, they normally reside in tissues and are involved in tissue homeostasis by participating in the remodeling, repair, and activation of certain metabolic functions. They can be activated by cytokines such as IL-4, IL-10, and IL-13, but also by more specific signals from the tissue environment [21].

The accumulation of immune cells, especially that of macrophages, as well as their inflammatory phenotype, affect adipose tissue homeostasis and, more specifically, the recruitment and function of adipocytes in white and brown adipose tissues [16]. It has been shown that TNFα secreted by M1 macrophages inhibited adipocyte differentiation [22] and that IL-1β blocked insulin signaling [23], thus favoring insulin-resistance. Recently, it has also been shown that IL-1β and TNFα can affect the thermogenic function of brown adipocyte [24,25,26]. These inflammatory cytokines thus participate in the deregulation of tissue homeostasis by limiting its ability to dissipate an excessive supply of substrate in the form of heat. On the contrary, it was shown that M2 macrophages, via the secretion of factors such as IL-4 or IL-13 favored the formation of brown adipocytes and their activation [27,28]. In addition, immune cells can modulate insulin sensitivity and local secretion of catecholamines [29]. This secretion, that represents the preferential inducer of lipolysis and thermogenesis through the activation of the β-adrenergic pathway, appears to be crucial during prolonged exposure to cold or aging [28,30].

Similarly to adipokines, the oxygenated derivatives of ω6 PUFAs such as the *n*-2 series prostaglandins or the *n*-4 series leukotrienes, which are synthesized and secreted by adipocytes, participate in the inflammatory state of the tissue [31,32]. Furthermore, adipocytes are able to metabolize ω3 PUFAs, in the same way as ω6, to produce oxygenated anti-inflammatory derivatives such as *n*-3 series prostaglandins (PG), *n*-5 series leukotrienes (LT), as well as resolvins (Rv) and protectins (PD) [32]. For example, the administration of ω3 PUFAs to obese mice as well as resolvin D1 (RvD1), an oxygenated derivative of DHA, limits macrophage infiltration, favors their polarization toward the M2 phenotype, and rescues adipocyte metabolic dysfunction [33,34]. Thus, ω6- and ω3-derived oxylipins are able to modulate the inflammatory phenotype of immune cells, especially macrophages [11,35]. As dietary ω6 and ω3 PUFAs directly affect the quality and the quantity of oxylipins synthesized and secreted by the adipocytes, it is of high interest to characterize the impact of ω3 PUFA diet supplementation on the inflammatory state of adipose tissue.

## 2. Materials and Methods 

### 2.1. Reagents

Culture media and buffer solutions were purchased from Lonza (Ozyme, St-Quentin en Yvelines, France), fetal bovine serum (FBS) from Eurobio (Courtaboeuf, France), insulin and trypsin from InVitrogen (Cergy Pontoise, France). Oxylipins and inhibitors were purchased from Cayman (BertinPharma, Montigny le Bretonneux, France). Other culture reagents were from Sigma-Aldrich Chimie (Saint-Quentin Fallavier, France). 

### 2.2. Animals and Diets

The experiments were conducted in accordance with the French and European regulations (Directive 2010/63/EU) for the care and use of research animals and were approved by national experimentation committees (MESR 01947.03). Ten-week-old C57BL/6J male mice from Janvier Laboratory (France) were maintained at thermoneutrality (28 ± 2 °C) and 12:12-h light-dark cycles, with ad libitum access to food and water to not hide any behavioral modification. Mice were fed for 12 weeks with isocaloric isoenergetic (3.2 kCal/g–13.5 kJ/g) ω6- or ω3-enriched diets (12% energy content as lipids). The diets were prepared by Harlan (WI, USA) from standard chow diets (reference number 2016) by the addition of specific fatty acid ethyl-esters from NuChekPrep (Elysian, MIN, USA). Detailed compositions are displayed in Table 1. Blood, interscapular brown adipose tissue (iBAT), epididymal (eWAT), and inguinal subcutaneous (scWAT) white adipose tissues were sampled and used for different analyses.

### 2.3. Cell Culture

THP-1, a human pro-monocytic cell line, was cultured in RPMI GlutaMax medium, supplemented with 10% FBS and 10 mM sodium pyruvate, at 37 °C and 5% CO_2_. Differentiation in macrophages-like cells was induced by treatment with 20 nmol/L phorbol 12-myristate 13-acetate (PMA) for 72 h. Then, media were replaced and polarization was induced for 48 h either with lipopolysaccharides (LPS, 100 ng/mL) for M1 like-phenotype or with IL-4/IL-10 (10 ng/mL each) for M2 like-phenotype acquisition. Treatments with a LOX inhibitor (=carnosic acid (CA), 10 µM), and/or with 9-HODE and 13-HODE (50 nmol/L + 50 nmol/L), were performed during the 48 h polarization step. 

### 2.4. Oxylipin Quantification

For quantification of unesterified oxylipins, tissues were snap-frozen with liquid nitrogen immediately after retrieval and stored at −80 °C. Extraction and analysis by mass spectrometry were performed at METATOUL platform (MetaboHUB, INSERM UMR 1048, I2MC, Toulouse, France) as previously described [13,36]. 

### 2.5. Cytokine Quantification

For blood analysis, plasmas were diluted twice and analysis following manufacturer’s instructions using the mouse V-PLEX Proinflammatory Panel 1 Kit (Meso Scale Discovery, # K15048D) on a QuickPlex SQ 120 apparatus (Meso Scale Discovery). 

For tissue analysis, proteins were extracted from frozen organs using an ULTRA TURRAX T25 (Ika, Germany) and lysis buffer (25 mM Tris-Cl (pH 7.4), 100 mM NaCl, 1 mM EDTA, 1% Triton X-100, 0.5% Nonidet P40 and protease inhibitors (Roche Diagnostics, Meylan, France)). Protein concentration was evaluated by BCA assay (Sigma-Aldrich Chimie, Saint-Quentin Fallavier, France). 10 µg proteins were used to evaluate cytokine concentration using the same kit and apparatus as those used for blood cytokine analysis. 

### 2.6. Histology

Freshly sampled tissues were fixed in 4% paraformaldehyde overnight at RT and then paraffin-embedded. Embedded tissues were cut into 5-μm sections and dried overnight at 37 °C. For immunohistochemistry, sections were then deparaffinized in xylene, rehydrated using alcohol, and washed in phosphate-buffered saline (PBS). 

For histology analysis, sections were stained with hematoxylin-eosin and mounted in Mowiol.

For immunohistochemistry analysis, antigen unmasking was performed in boiling citrate buffer (10 mM, pH 6.0) for 6 minutes. Sections were then permeabilized in PBS with 0.2% Triton X-100 at room temperature for 20 minutes and blocked in the same buffer containing 3% BSA for 30 min. Sections were co-incubated with rat anti-F4/80 antibody (Biorad, clone Cl:A3-1, dilution 1:100) and rabbit anti-Arginase-1 (ThermoFisher Scientific, #PA5-29645, dilution 1:100) overnight at 4 °C. 

Following a 30-min incubation with biotinylated anti-rat and TRITC-coupled anti-rabbit secondary antibodies, the sections were incubated for another 30 min at room temperature with avidin–biotin complex (Vector Lab, VECTASTAIN ABC Kit, PK-4000), and were then labeled with 3,3′-diaminobenzidine solution (Vector Lab, DAB, SK-4100). Nuclear staining was performed with DAPI and sections were mounted in Mowiol. 

Visualization was performed with an Axiovert microscope. Pictures were captured using AxioVision software (Carl Zeiss, Jena, Germany).

### 2.7. Isolation and Analysis of RNA

Procedures follow MIQE recommendations [37]. Total RNA was extracted using a TRI-Reagent kit (Euromedex, Souffelweyersheim, France) according to the manufacturer’s instructions. For RNA isolation from organs, tissues were homogenized in TRI-Reagent using a dispersing instrument (ULTRA TURRAX T25). A reverse transcription-polymerase chain reaction (RT-PCR) was performed using M-MLV-RT (Promega). SYBR qPCR premix Ex TaqII from Takara (Ozyme, France) was used for quantitative PCR (qPCR), and assays were run on a StepOne Plus ABI real-time PCR machine (PerkinElmer Life and Analytical Sciences, Boston). The expression of selected genes was normalized to that of the TATA-box binding protein (TBP) and 36B4 housekeeping genes and then quantified using the comparative-ΔCt method. Primer sequences are available upon request.

### 2.8. Statistical Analysis

Data were expressed as mean values ± standard error of the mean (SEM). Data were analyzed using InStat software (GraphPad Software) by one-way ANOVA followed by a Mann-Whitney (for in vivo experiments) or a Student-Newman-Keuls (for in vitro experiments) post-test to assess statistical differences between experimental groups. Differences were considered statistically significant with *p* < 0.01.

## 3. Results

### 3.1. Impact of ω3 PUFA Supplementation on General Parameters of Mice

#### 3.1.1. General Metabolic Parameters

Ten-week-old male mice were fed for 12 weeks with an isocaloric isoenergetic standard diet enriched in ω6 PUFAs (ω6-enriched diet, *ω6/ω3* = 30), or supplemented with ω3 PUFAs (ω3-enriched diet, *ω6/ω3* = 3.7), see Table 1. Mice were housed at 28 °C, near thermoneutrality, in order to limit energy expenditure due to thermogenic metabolism and to avoid any effect of this activity on inflammatory response, as demonstrated previously [38]. 

**Figure 1:**
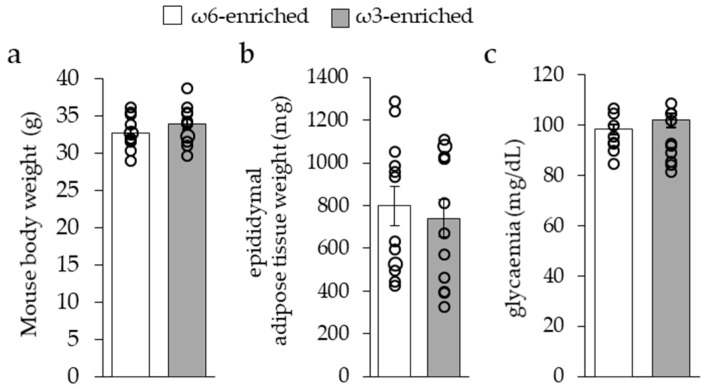
Mice general metabolic parameters. (**a**) Mouse body weight, (**b**) epididymal white adipose tissue weight, and (**c**) blood glycaemia evaluated after 12 weeks of ω6- or ω3-enriched diet. Results are displayed as independent mouse values (dots) and mean ± SEM (histograms). *n* = 12.

Mice body weight, see Figure 1a, as well as food intake (ω6-enriched diet, 4.49 g/day; ω3-enriched diet, 4.46 g/day per mouse) were similar between the two groups. Epididymal white adipose tissue mean weight, see Figure 1b, and fed glycaemia, see Figure 1c, were not different after 12 weeks of the diets. Altogether, these results indicated that the *ω6/ω3* ratio of a standard diet, equilibrated in carbohydrate, protein, and fat quantities (respectively, 20.1%, 65.4%, and 14.5% of energy supply), did not modify general metabolic parameters of mice.

#### 3.1.2. Plasmatic Inflammatory Phenotype

**Figure 2:**
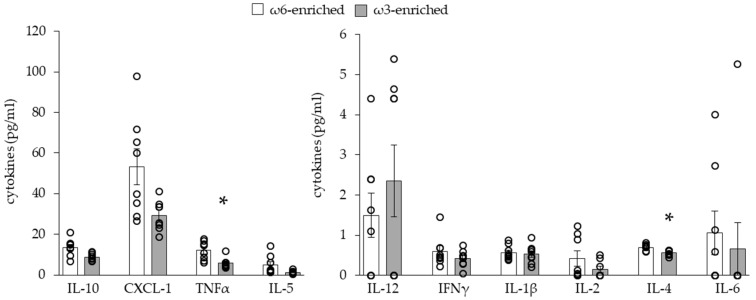
Analysis of blood cytokines. Results are displayed as independent mouse values (dots) and mean ± SEM (histograms). *n* = 8. *, *p* < 0.01.

To characterize the systemic inflammatory effect of a PUFA-enriched diet, we evaluated the blood circulating level of a panel of cytokines, see Figure 2. 

As expected, the level of most of the pro-inflammatory and anti-inflammatory cytokines was unchanged between the two groups of mice. Only TNFα (pro-inflammatory cytokine) and IL-4 (anti-inflammatory cytokine) levels slightly but significantly decreased in mice fed an ω3-enriched diet. 

#### 3.1.3. Impact of ω3 PUFA Supplementation on Adipose Tissue Oxylipin Content

**Figure 3:**
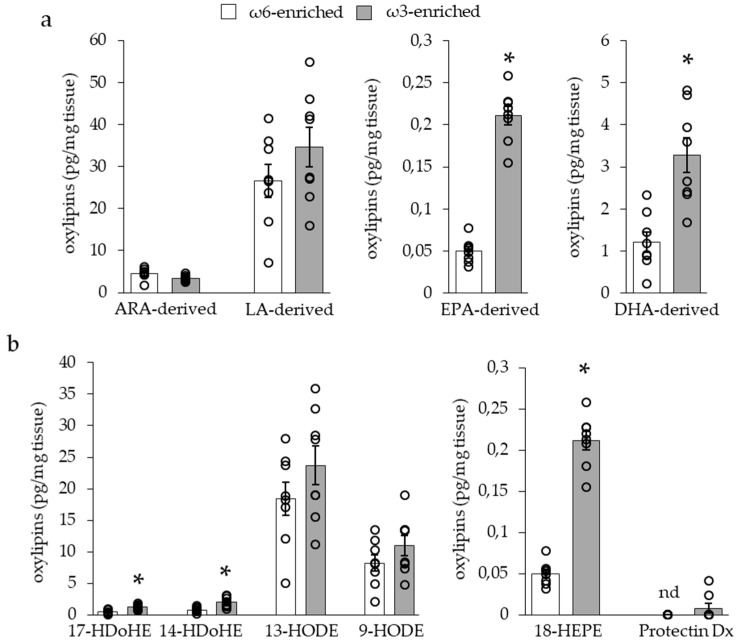
Quantities of oxylipins derived from dietary polyunsaturated fatty acids (PUFAs) in interscapular brown adipose tissue (iBAT). (**a**) Quantities of oxylipins derived from arachidonic acid (ARA) and linoleic acid (LA) ω6 PUFAS or eicosapentaenoic acid (EPA) and docosahexaenoic acid (DHA) ω3 PUFAs. (**b**) Quantities of oxylipins considered as anti-inflammatory or pro-resolving mediator intermediates. Results are displayed as independent mouse values (dots) and mean ± SEM (histograms). *n* = 8. *, *p* < 0.01.

**Figure 4:**
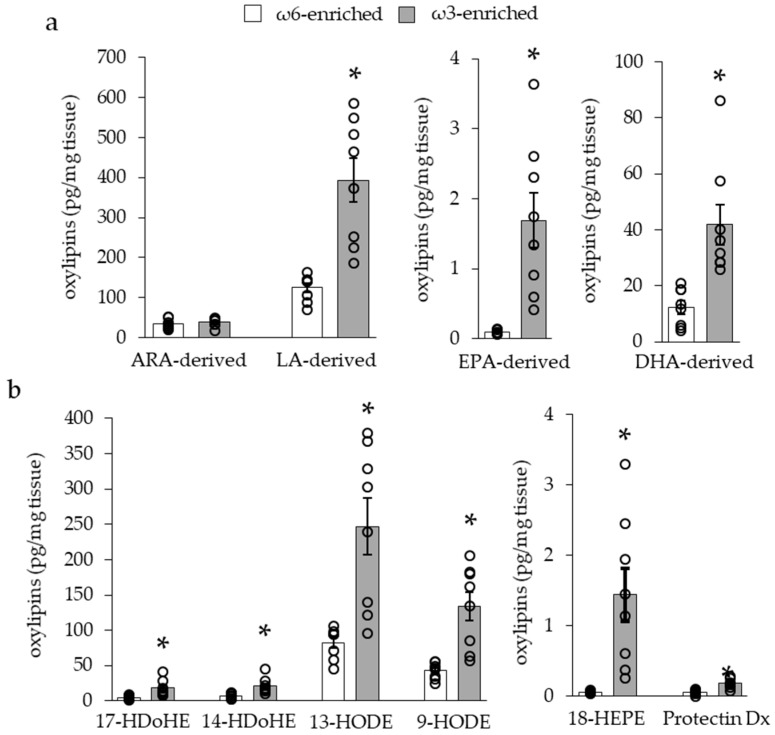
Quantities of oxylipins derived from dietary PUFAs in inguinal subcutaneous white adipose tissues (scWAT). (**a**) Quantities of oxylipins derived from ARA and LA ω6 PUFAS or EPA and DHA ω3 PUFAs. (**b**) Quantities of oxylipins considered as anti-inflammatory or pro-resolving mediator intermediates. Results are displayed as independent mouse values (dots) and mean ± SEM (histograms). *n* = 8. *, *p* < 0.01.

To investigate the modification induced by the two different diets within adipose tissues, we quantified the levels of 33 PUFA-metabolites within iBAT, see Figure 3, and scWAT, see Figure 4, of mice. These oxylipins were analyzed by groups following their PUFA origin, see Figure 3a and Figure 4a, or separately, see Figure 3b and Figure 4b. In the iBAT, ω3 PUFA supplementation led to a significant increase of the oxylipins deriving from ω3 PUFAs EPA (PGE3, LTB5, 18-HEPE) and DHA (RvD2, RvD1, MaR1, PDx, 17-HDoHE, 14-HDoHE), but did not affect ω6-derived metabolites (6kPGF1a, TxB2, 11B-PGF2a, PGF2a, PGE2, PGD2, 8isoPGA2, 15dPGJ2, LxB4, LxA4, LTB4, 5,6-DiHETE, 15-HETE, 8-HETE, 12-HETE, 5-HETE, 5oxoETE, 14,15-EET, 11,12-EET, 8,9-EET, 5,6-EET derived from ARA; 13-HODE, 9-HODE derived from LA), see Figure 3a. 

In scWAT, while similar results were found for ω3 PUFA-derived and ARA-derived oxylipins, LA-derived metabolites were highly increased, as shown in Figure 4a. 

LA and ω3-PUFA derived oxylipins are considered as anti-inflammatory and pro-resolving mediators, especially through the modulation of macrophage function. Along with these oxylipins, we have found that 14- and 17-HDoHEs and 18-HEPE levels were increased in iBAT and scWAT of mice fed the ω3-enriched diet, and 9- and 13-HODEs were increased only in scWAT, see Figure 3b and Figure 4b. 14- and 17-HDoHE are metabolized in pro-resolving mediators as RvD1, RvD2, Mar1, PDx, and PD1, while 18-HEPE leads to RvE1 synthesis. It is interesting to note that these final metabolites were barely (PDx) or not detected within the tissue, see Figure 3b and Figure 4b. 

### 3.2. Effect on Inflammatory Phenotype of Adipose Tissue 

#### 3.2.1. Histology and Cytokine Content

**Figure 5:**
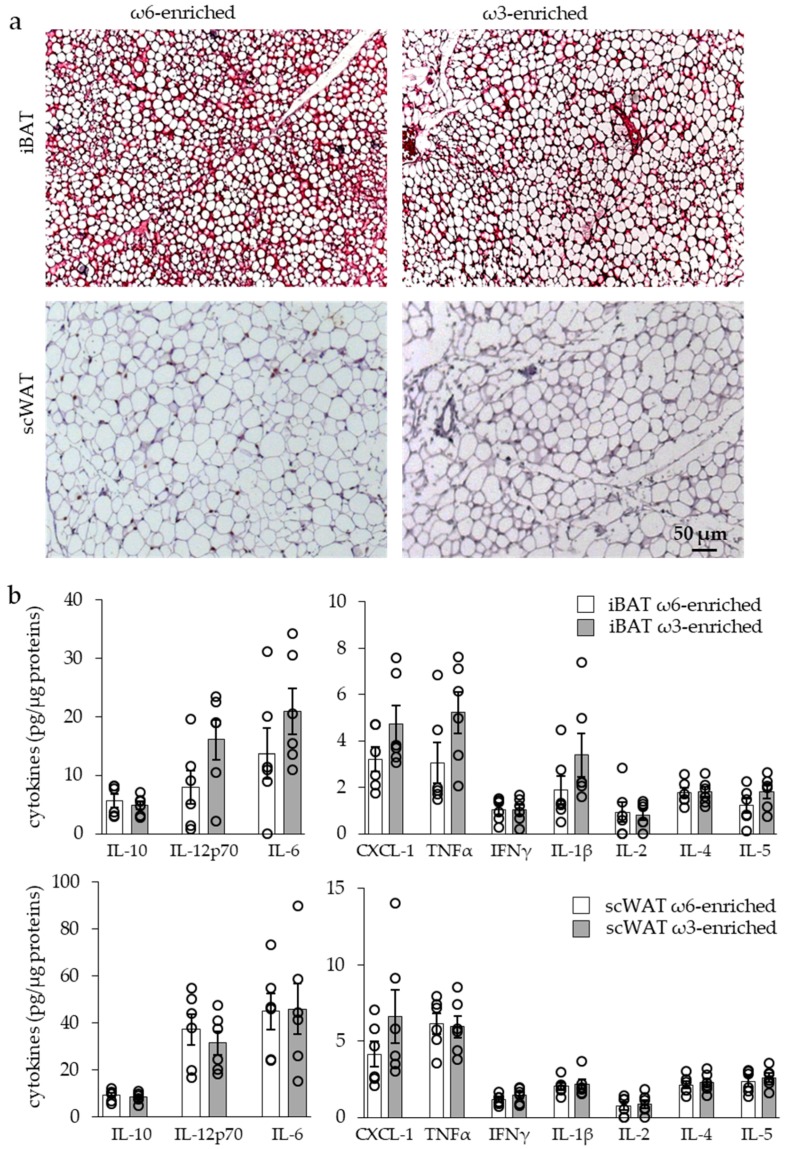
Inflammatory profile of iBAT and scWAT of mice submitted to ω6- or ω3-enriched diet. (**a**) Hematoxylin and eosin staining of tissue sections. (**b**) Analysis of adipose tissue cytokine levels. Results are displayed as independent mouse values (dots) and mean ± SEM (histograms). *n* = 6. *, *p* < 0.01.

The histological analysis of iBAT and scWAT, see Figure 5a, revealed neither cell infiltration nor crown structure that were typical of an adipose tissue inflammatory response in both groups of mice. 

In the same way, analysis of the iBAT and scWAT cytokine contents showed similar levels of both pro- and anti-inflammatory cytokines in the two groups of mice, as shown in Figure 5b.

#### 3.2.2. Expression of Inflammatory Markers

**Figure 6:**
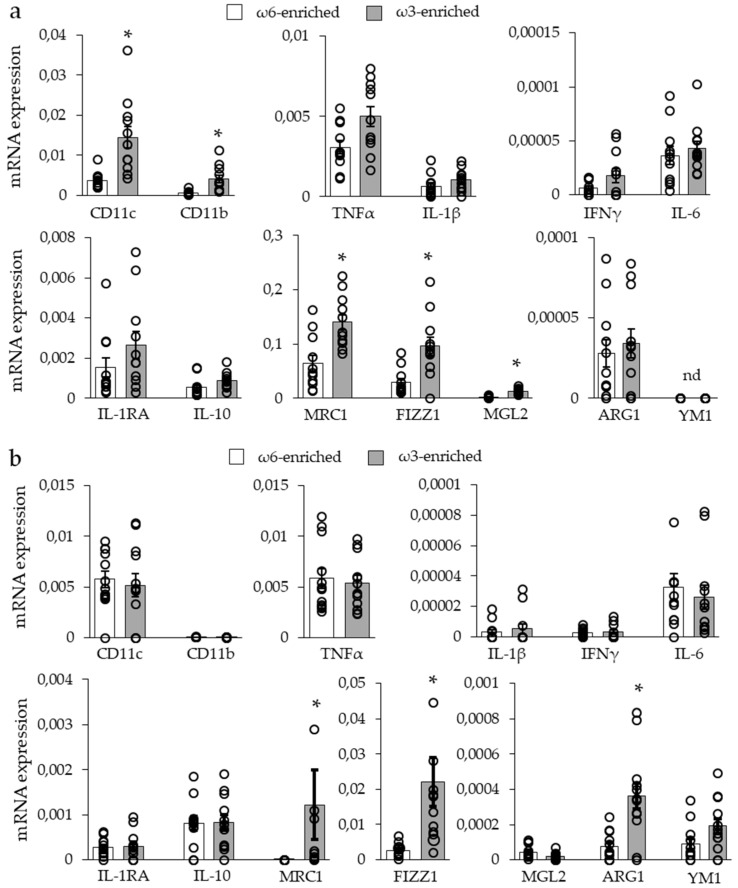
Macrophage marker expression in adipose tissue of mice submitted to ω6- or ω3-enriched diet. mRNA level analysis of general (CD11c, CD11b), M1 (TNFα, IL-1β, IFNγ, IL-6) and M2 (IL-1RA, IL-10, MRC1, FIZZ1, MGL2, ARG1, YM1) macrophage markers in (**a**) iBAT and (**b**) scWAT. Histograms display mean ± SEM. *n* = 12. *, *p* < 0.01.

As we did not find any modulation of cytokine levels, we analyzed marker expression of specialized macrophages to evaluate the inflammatory background of the tissue, see Figure 6. 

The analysis of macrophage markers in iBAT derived from the ω3-enriched diet group, see Figure 6a, revealed an increase in CD11b (or ITGAM, integrin αM) and CD11c (or ITGAX, integrin αX) mRNA expression, concomitantly to an increase in major M2 macrophage markers, namely MRC1 (mannose receptor 1), FIZZ1 (found in inflammatory zone 1 or RELMα), and MGL2 (macrophage galactose N-acetyl-galactosamine specific lectin 2). No change was found for other M2 macrophage markers or for M1 macrophage markers. To note, ARG1 (arginase 1) and Ym1 (chitinase 3-like 3) were either barely detected or undetected in this tissue. 

The analysis of the scWAT, see Figure 6b, from ω3-supplemented mice, showed an increased the expression of the M2 macrophage markers MRC1 and FIZZ1 (not MGL2), but no increase of CD11c (CD11b was undetected). In contrast to iBAT, our data revealed an increase of ARG1 mRNA expression and the induction of YM1 mRNA expression. Finally, as for iBAT, no change was found for mRNA expression of M1 macrophage markers. 

Altogether, these results demonstrated that an ω3-enriched diet led to a general increase in M2 anti-inflammatory macrophage marker expression without modification in M1 pro-inflammatory markers. This was correlated and perhaps due to the increased amount of substrates for pro-resolving mediator synthesis, as well as an increased quantity of M2 polarizing oxylipins.

### 3.3. Effect of Potential Anti-Inflammatory Oxylipins Modified in an ω3-Enriched Diet on THP1 Monocyte Cells 

**Figure 7:**
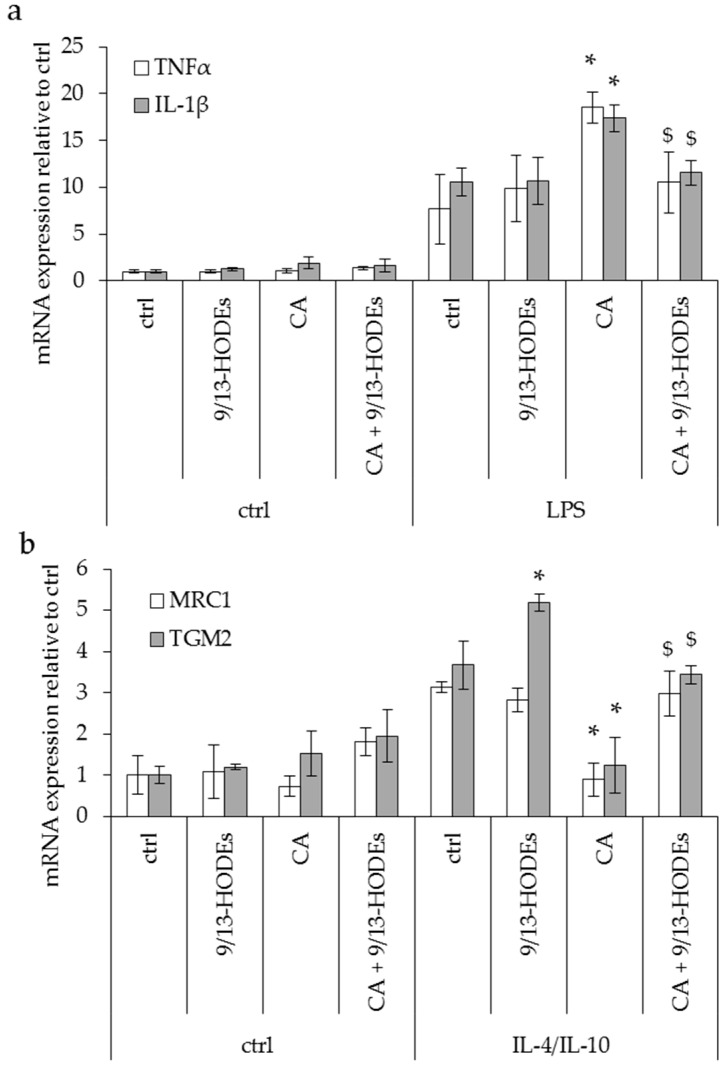
Macrophage marker expression in THP-1 cells under 9- and 13-HODE treatment. mRNA level analysis by RT-qPCR of M1 (TNFα, IL-1β) and M2 (MRC1, TGM2) macrophage markers in control, lipopolysaccharides (LPS) (upper panel) or IL-4/IL-10 (lower panel) treated THP-1 macrophages. Cells were co-treated for 48 h with carnosic acid (CA, 10 µM) and or 9- and 13-HODEs. (**a**) CA treatment induced opposite effects in M1- and M2-like macrophages as it increased inflammatory markers in THP-1 M1-like macrophages, and (**b**) decreased M2-like macrophages’ markers. Histograms display mean ± SEM. *n* = 3. *, *p* < 0.01 vs. ctrl and $, *p* < 0.01 vs. CA.

The oxylipins 9- and 13-HODEs (LA-derived oxylipins metabolized by LOX) are not known to be precursors of pro-resolving mediators but display high contents in iBAT, see Figure 3b, and scWAT, see Figure 4b, and are strongly increased in scWAT after the implementation of an ω3-enriched diet. In order to investigate the role of 9- and 13-HODEs on macrophage polarization, we used THP-1 macrophage cell lines activated in pro-inflammatory M1 (LPS 100 ng/mL, Figure 7a) or anti-inflammatory M2-like phenotype (IL4 + IL-10 10 ng/mL each, Figure 7b). THP-1 cells were treated with 9- and 13-HODEs (9/13-HODEs, 50 nmol/L each) or with carnosic acid (CA, 10 µM), a lipoxygenase inhibitor [39], or a combination of both. 

None of the treatments modulated non-polarized THP-1, see Figure 7. Treatment with 9/13-HODEs alone showed no effect on macrophages’ M1-like phenotype but increased TGM2 expression on M2-like macrophages. Remarkably, CA treatment induced opposite effects in M1- and M2-like macrophages as it increased inflammatory markers in THP-1 M1-like macrophages, see Figure 7a, and decreased M2-like macrophages’ markers, see Figure 7b. Interestingly, 9/13-HODEs supplementation reversed CA effects, see Figure 7.

## 4. Discussion

Dietary fats are the source of essential PUFAs that are required for fetal and newborn development and trigger a variety of biological responses in adults, especially, in adipose tissue. New dietary recommendations warn against the insufficient intake of ω3 PUFAs and the excess of ω6 PUFAs which correlate with various disease developments [3,4]. In the first year of life, a high dietary *ω6/ω3* ratio is positively associated with adiposity of infants [40,41,42]. In the same way, in adults, a high *ω6/ω3* ratio can correlate to an increase of fat mass and the development of metabolic complications [6,7,8,9,10]. 

Conversely, it has been described that a low *ω6/ω3* ratio seems to be correlated with metabolic disorder protection in different populations [43]. On a metabolic point of view, diets exhibiting a high *ω6/ω3* ratio allow a higher ARA bioavailability for the synthesis of ω6-derived eicosanoids due to an insufficient compensatory effect of EPA and DHA [15]. Indeed, both ω6 and ω3 PUFAs are metabolized using the same enzymatic pathways. First, LA and LNA are modified by common Δ-desaturases and elongases [44]; then, their metabolites, i.e., ARA, DGLA, EPA, and DHA, are metabolized in oxygenated derivatives also using common pathways involving cyclooxygenases, lipoxygenases, and CYP450 enzymatic reactions. Here, we provided evidence that, compared to a high *ω6/ω3* PUFA ratio, an equilibrated ratio of four allows the synthesis of LA and EPA/DHA oxylipins instead of ARA oxylipins. As LA and LNA use a common pathway (Δ-desaturase) to be transformed, respectively, into DGLA/ARA and EPA/DHA, we hypothesize that LNA supplementation could limit LA desaturation and thus increase LA bioavailability and metabolization in oxylipins through the LOX pathways. Thus, these competitive phenomena, in addition to dietary intake, determine PUFA availability in oxylipins synthesis and, in turn, their various metabolic effects, especially for inflammatory responses [45]. 

It has already been described in rodents that an increase of white adipose tissue mass can be related to an ω6 PUFA-enriched high-fat diet and can be prevented by ω3 PUFA supplementation [12,46]. It is suggested that this could only be due to a specific subset of ω3 PUFA such as EPA [47]. Moreover, eicosanoids derived from ω6 PUFA inhibit adipocyte thermogenic activity both in vitro and in vivo [11,13,48]. We and others demonstrated previously, using the same nutritional approach as in the present work, that an ω3 PUFA diet supplementation improved the thermogenic adipocyte function by promoting a more oxidative phenotype in response to β-adrenergic stimulation [14,49]. In the present study, ω3 PUFA supplementation does not induce any change in body mass, glycaemia, or white and brown adipose tissue morphologies since the mice were fed diets with normal fat content and did not receive any β-adrenergic challenge.

Most studies concerning ω3 PUFA supplementations were carried out in a context of obesity (high-fat diet) or infection (LPS treatment) and demonstrate a positive effect of ω3 PUFA supplementation on the analyzed parameters [35]. Nevertheless, other studies demonstrate the inability of ω3 PUFAs to modulate inflammation after LPS treatment [50] or in obese mice [51,52]. These discrepancies are essentially due to the differences in the experimental approaches (diet composition, mouse strain, challenge….) and in the analyzed parameters (cytokine concentration, mRNA expression, histology…). In humans, several experimental approaches have tried to link an ω3 PUFA intake to inflammatory response, again with inconsistent conclusions. For example, a one-year dietary supplementation in ω3 PUFA does not modify the circulating cytokine levels in healthy volunteers [53]. Conversely, other studies show a decrease of blood inflammatory markers after ω3 PUFA supplementation [54,55]. It is important to note that a plasma inflammatory mediator profile seems to be less representative compared to the one of adipose tissue [56]. The same discrepancy is found for studies analyzing adipose tissue inflammation. Although one human trial (4g ω3 PUFAs/day; 12 weeks) on insulin-resistant adults demonstrates a decrease in the crown-like structure number [57], corresponding to phagocytic activity of macrophage on adipocyte, another trial on the same type of patients (4.2g ω3 PUFAs/day; 6 months) demonstrates no effect of ω3 PUFA supplementation on the same parameter [58]. Moreover, a recent paper establishes that the oxylipin profile in rat adipose tissue after dietary ω3 PUFA supplementation (ratio *ω6/ω3* of 0.6) is dependent of (i) the kind of ω3 PUFA used, (ii) the kind of adipose tissue analyzed, and (iii) the sex [59].

In view of these heterogeneities, we decided to analyze the effect of PUFA intake in normal physiological conditions (thermoneutrality, no β-adrenergic challenge) using an isocaloric, isoenergetic standard diet supplemented with ethyl esters of fatty acids (instead of classic oil supplementation) and various technical approaches to characterize the inflammatory profile. With this strategy, we characterize fatty acid metabolism within subcutaneous and brown adipose tissues and the related inflammatory phenotype. Our results linking ω3 PUFA supplementation and M2 macrophage are in line with other studies, such as a recent one demonstrating that (i) treatment of human adipose tissue explants with ω3 PUFAs lead to an anti-inflammatory phenotype characterized by a decrease of M1 marker expression, and (ii) treatment of THP-1 cells increased expression of M2 markers [60]. In the same way, DHA supplementation in a high-fat diet context promotes mRNA expression of M2 markers within white adipose tissue without affecting the total macrophage number [61]. In this study, the authors describe the same effect for RvD1, DHA metabolites, and conclude that DHA leads to an anti-inflammatory phenotype via RvD1 synthesis. Unfortunately, they never quantify RvD1 production in vivo and thus do not link DHA supplementation to RvD1 synthesis [61]. In our study, we have not been able to detect resolvins but only their substrates. We assume that without a specific inflammatory signal, intermediates of pro-resolving mediators are synthesized but not metabolized. Indeed, these mediators are involved in the resolution of inflammation and appeared late in the process as they are not required before, differently to prostaglandins and leukotrienes which appear early [62]. 

In our study, we measure a defined set of oxylipins. Even if this panel includes oxylipins deriving from all pathways and PUFAs, we cannot exclude that unmeasured oxylipins triggered the anti-inflammatory effect of ω3 PUFA supplementation found in our model. In this way, epoxide and diol metabolites derived from CYP epoxygenase/soluble epoxide hydrolase activity [63], as well as endocannabinoids, are known and interesting potential mediators of the inflammatory effect of PUFA [64]. In addition, the esterification of oxylipins, especially of eicosanoids, was described as an active and major mechanism in various cell biological responses including inflammation [65]. These esterified oxylipins can represent the majority of cell oxylipins and can be hydrolyzed from the membrane under specific stimuli [66]. In this way, it could be interesting to quantify all oxylipins (unesterified and esterified) in adipose tissue under ω3 PUFA diet supplementation and to evaluate their hydrolysis under inflammatory conditions. Nevertheless, our unexhaustive analysis allowed a correlation between the synthesis of several oxylipins and the expression of M2 macrophage markers. We propose that 9- and 13-HODEs could drive this effect. In our in vitro results on the THP-1 cell line, we demonstrate that 9- and 13-HODEs are not enough to directly drive the polarization of THP-1 macrophage but are required to maintain the phenotype. Indeed, their supplementation restores control level expression of M1-like and M2-like markers after CA treatment. Moreover, 9- and 13-HODEs seem to play a role in the anti-inflammatory effects since they are able to increase M2 markers such as TGM2. These results are consistent with some studies describing 9- and 13-HODEs as known mediators of macrophage polarization [67] in a PPARγ-dependent manner [68]. Of course, other oxylipins could be involved in the anti-inflammatory environment found in our mice. For example, the study of Fat-1 mouse, which is able to synthesize ω3 PUFAs itself, displays a lowered inflammatory environment induced by obesity, correlatively to 17-HDoHE synthesis [69]. In addition to oxylipins involvement, we cannot exclude a direct action of ω3 PUFAs on the membrane receptor. Indeed, it is shown that DHA is able to directly activate, via GPR120, an anti-inflammatory response driven by macrophage within adipose tissue [33]. This activity could be linked to the recent characterization of the DHA inhibitory effect on NLRP3 inflammasome activity, an effect triggered by GPR40/GPR120 pathways and leading to a decreased production of mature IL-1β [70]. As NLRP3 is activated essentially in response to an infectious environment, we do not correlate ω3 PUFA supplementation with a decrease in IL-1β production in our physiological context.

It is interesting to note that the ω3 PUFA intake finely drives the kind of oxylipins synthesized. A recent study analyzed the effect of an ω3 PUFA dietary supplementation of an already equilibrated diet (ratio *ω6/ω3* = 6.7) to reach an *ω6/ω3* ratio of 0.8. Thus, differently to our situation, LA and LNA are already desaturated equivalently, and the increase in ω3 PUFA intake leads to a decrease of LA-derived oxylipins (9/13-HODEs) in favor of EPA and DHA derived oxylipins in the brain. Moreover, this “over”-supplementation ameliorates against an inflammatory response [71].

## 5. Conclusions

Previous studies have demonstrated the positive effect of ω3 PUFA intake to counteract the adverse consequences of a high-fat diet or inflammatory situation. Herein, our study was conducted in non-obesogenic non-inflammatory conditions and also showed a beneficial influence of ω3 PUFA dietary supplementation on the adipose tissue inflammatory phenotype. Moreover, while ω3 PUFA metabolites have been involved in this effect, we additionally highlighted the unsuspected role of LA-derived metabolites. Finally, this already assumed beneficial outcome of ω3 PUFA supplementation is in line with a human situation where a high *ω6/ω3* ratio is correlated with the development of inflammatory diseases in metabolic tissue. 

## Figures and Tables

**Table 1:** Diet compositions.

	ω6-Enriched Diet	ω3-Enriched Diet
Protein [% by weight]	16	
Carbohydrate [% by weight]	52	
Fat [% by weight]	5	
Saturated fatty acids (FAs) [% of total FAs]	12	
Monounsaturated FAs [% of total FAs]	26	14
Polyunsaturated FAs [% of total FAs]	62	74
Linoleic acid [% by weight]	3	
α-linolenic acid [% by weight]	0.1	0.64
EPA [% by weight]	-	0.08
DHA [% by weight]	-	0.08
*ω6/ω3* PUFA ratio	30	3.75

## References

[1] Simopoulos A.P. (2002). The importance of the ratio of omega-6/omega-3 essential fatty acids. Biomed. Pharmacother..

[2] Simopoulos A.P. (2016). An Increase in the Omega-6/Omega-3 Fatty Acid Ratio Increases the Risk for Obesity. Nutrients.

[3] Ailhaud G., Massiera F., Weill P., Legrand P., Alessandri J.M., Guesnet P. (2006). Temporal changes in dietary fats: Role of *n*-6 polyunsaturated fatty acids in excessive adipose tissue development and relationship to obesity. Prog. Lipid Res..

[4] Muhlhausler B.S., Ailhaud G.P. (2013). Omega-6 polyunsaturated fatty acids and the early origins of obesity. Curr. Opin. Endocrinol. Diabetes Obes..

[5] Simopoulos A.P., DiNicolantonio J.J. (2016). The importance of a balanced omega-6 to omega-3 ratio in the prevention and management of obesity. Open Heart.

[6] Inoue K., Kishida K., Hirata A., Funahashi T., Shimomura I. (2013). Low serum eicosapentaenoic acid/arachidonic acid ratio in male subjects with visceral obesity. Nutr. Metab. (Lond.).

[7] Savva S.C., Chadjigeorgiou C., Hatzis C., Kyriakakis M., Tsimbinos G., Tornaritis M., Kafatos A. (2004). Association of adipose tissue arachidonic acid content with BMI and overweight status in children from Cyprus and Crete. Br. J. Nutr..

[8] Williams E.S., Baylin A., Campos H. (2007). Adipose tissue arachidonic acid and the metabolic syndrome in Costa Rican adults. Clin. Nutr..

[9] Claria J., Nguyen B.T., Madenci A.L., Ozaki C.K., Serhan C.N. (2013). Diversity of lipid mediators in human adipose tissue depots. Am. J. Physiol. Cell Physiol..

[10] Garaulet M., Perez-Llamas F., Perez-Ayala M., Martinez P., de Medina F.S., Tebar F.J., Zamora S. (2001). Site-specific differences in the fatty acid composition of abdominal adipose tissue in an obese population from a Mediterranean area: Relation with dietary fatty acids, plasma lipid profile, serum insulin, and central obesity. Am. J. Clin. Nutr..

[11] Ghoshal S., Trivedi D.B., Graf G.A., Loftin C.D. (2011). Cyclooxygenase-2 deficiency attenuates adipose tissue differentiation and inflammation in mice. J. Biol. Chem..

[12] Massiera F., Saint-Marc P., Seydoux J., Murata T., Kobayashi T., Narumiya S., Guesnet P., Amri E.Z., Negrel R., Ailhaud G. (2003). Arachidonic acid and prostacyclin signaling promote adipose tissue development: A human health concern?. J. Lipid Res..

[13] Pisani D.F., Ghandour R.A., Beranger G.E., Le Faouder P., Chambard J.C., Giroud M., Vegiopoulos A., Djedaini M., Bertrand-Michel J., Tauc M. (2014). The omega6-fatty acid, arachidonic acid, regulates the conversion of white to brite adipocyte through a prostaglandin/calcium mediated pathway. Mol. Metab..

[14] Ghandour R.A., Colson C., Giroud M., Maurer S., Rekima S., Ailhaud G.P., Klingenspor M., Amri E.Z., Pisani D.F. (2018). Impact of dietary omega3 polyunsaturated fatty acid supplementation on brown and brite adipocyte function. J. Lipid Res..

[15] Fischer R., Konkel A., Mehling H., Blossey K., Gapelyuk A., Wessel N., von Schacky C., Dechend R., Muller D.N., Rothe M. (2014). Dietary omega-3 fatty acids modulate the eicosanoid profile in man primarily via the CYP-epoxygenase pathway. J. Lipid Res..

[16] Odegaard J.I., Chawla A. (2013). The immune system as a sensor of the metabolic state. Immunity.

[17] Tilg H., Moschen A.R. (2006). Adipocytokines: Mediators linking adipose tissue, inflammation and immunity. Nat. Rev. Immunol..

[18] Hotamisligil G.S. (2006). Inflammation and metabolic disorders. Nature.

[19] Lumeng C.N., Bodzin J.L., Saltiel A.R. (2007). Obesity induces a phenotypic switch in adipose tissue macrophage polarization. J. Clin. Investig..

[20] Sica A., Mantovani A. (2012). Macrophage plasticity and polarization: In vivo veritas. J. Clin. Investig..

[21] Roszer T. (2015). Understanding the Mysterious M2 Macrophage through Activation Markers and Effector Mechanisms. Mediat. Inflamm..

[22] Hotamisligil G.S., Shargill N.S., Spiegelman B.M. (1993). Adipose expression of tumor necrosis factor-alpha: Direct role in obesity-linked insulin resistance. Science.

[23] Jager J., Gremeaux T., Cormont M., Le Marchand-Brustel Y., Tanti J.F. (2007). Interleukin-1beta-induced insulin resistance in adipocytes through down-regulation of insulin receptor substrate-1 expression. Endocrinology.

[24] Okla M., Zaher W., Alfayez M., Chung S. (2018). Inhibitory Effects of Toll-Like Receptor 4, NLRP3 Inflammasome, and Interleukin-1beta on White Adipocyte Browning. Inflammation.

[25] Goto T., Naknukool S., Yoshitake R., Hanafusa Y., Tokiwa S., Li Y., Sakamoto T., Nitta T., Kim M., Takahashi N. (2016). Proinflammatory cytokine interleukin-1beta suppresses cold-induced thermogenesis in adipocytes. Cytokine.

[26] Sakamoto T., Takahashi N., Sawaragi Y., Naknukool S., Yu R., Goto T., Kawada T. (2013). Inflammation induced by RAW macrophages suppresses UCP1 mRNA induction via ERK activation in 10T1/2 adipocytes. Am. J. Physiol. Cell Physiol..

[27] Lee Y.H., Kim S.N., Kwon H.J., Maddipati K.R., Granneman J.G. (2016). Adipogenic role of alternatively activated macrophages in beta-adrenergic remodeling of white adipose tissue. Am. J. Physiol. Regul. Integr. Comp. Physiol..

[28] Qiu Y., Nguyen K.D., Odegaard J.I., Cui X., Tian X., Locksley R.M., Palmiter R.D., Chawla A. (2014). Eosinophils and type 2 cytokine signaling in macrophages orchestrate development of functional beige fat. Cell.

[29] Bolus W.R., Hasty A.H. (2018). Contributions of Innate Type 2 Inflammation to Adipose Function. J. Lipid Res..

[30] Camell C.D., Sander J., Spadaro O., Lee A., Nguyen K.Y., Wing A., Goldberg E.L., Youm Y.H., Brown C.W., Elsworth J. (2017). Inflammasome-driven catecholamine catabolism in macrophages blunts lipolysis during ageing. Nature.

[31] Hardwick J.P., Eckman K., Lee Y.K., Abdelmegeed M.A., Esterle A., Chilian W.M., Chiang J.Y., Song B.J. (2013). Eicosanoids in metabolic syndrome. Adv. Pharmacol..

[32] Masoodi M., Kuda O., Rossmeisl M., Flachs P., Kopecky J. (2015). Lipid signaling in adipose tissue: Connecting inflammation & metabolism. Biochim. Biophys. Acta.

[33] Oh D.Y., Talukdar S., Bae E.J., Imamura T., Morinaga H., Fan W., Li P., Lu W.J., Watkins S.M., Olefsky J.M. (2010). GPR120 is an omega-3 fatty acid receptor mediating potent anti-inflammatory and insulin-sensitizing effects. Cell.

[34] Titos E., Claria J. (2013). Omega-3-derived mediators counteract obesity-induced adipose tissue inflammation. Prostaglandins Other Lipid Mediat..

[35] Liddle D.M., Hutchinson A.L., Wellings H.R., Power K.A., Robinson L.E., Monk J.M. (2017). Integrated Immunomodulatory Mechanisms through which Long-Chain *n*-3 Polyunsaturated Fatty Acids Attenuate Obese Adipose Tissue Dysfunction. Nutrients.

[36] Le Faouder P., Baillif V., Spreadbury I., Motta J.P., Rousset P., Chene G., Guigne C., Terce F., Vanner S., Vergnolle N. (2013). LC-MS/MS method for rapid and concomitant quantification of pro-inflammatory and pro-resolving polyunsaturated fatty acid metabolites. J. Chromatogr. B Analyt. Technol. Biomed. Life Sci..

[37] Bustin S.A., Benes V., Garson J.A., Hellemans J., Huggett J., Kubista M., Mueller R., Nolan T., Pfaffl M.W., Shipley G.L. (2009). The MIQE guidelines: Minimum information for publication of quantitative real-time PCR experiments. Clin. Chem..

[38] Tian X.Y., Ganeshan K., Hong C., Nguyen K.D., Qiu Y., Kim J., Tangirala R.K., Tontonoz P., Chawla A. (2016). Thermoneutral Housing Accelerates Metabolic Inflammation to Potentiate Atherosclerosis but Not Insulin Resistance. Cell Metab..

[39] Poeckel D., Greiner C., Verhoff M., Rau O., Tausch L., Hornig C., Steinhilber D., Schubert-Zsilavecz M., Werz O. (2008). Carnosic acid and carnosol potently inhibit human 5-lipoxygenase and suppress pro-inflammatory responses of stimulated human polymorphonuclear leukocytes. Biochem. Pharmacol..

[40] Donahue S.M., Rifas-Shiman S.L., Gold D.R., Jouni Z.E., Gillman M.W., Oken E. (2011). Prenatal fatty acid status and child adiposity at age 3 y: Results from a US pregnancy cohort. Am. J. Clin. Nutr..

[41] Moon R.J., Harvey N.C., Robinson S.M., Ntani G., Davies J.H., Inskip H.M., Godfrey K.M., Dennison E.M., Calder P.C., Cooper C. (2013). Maternal plasma polyunsaturated fatty acid status in late pregnancy is associated with offspring body composition in childhood. J. Clin. Endocrinol. Metab..

[42] Rudolph M.C., Young B.E., Lemas D.J., Palmer C.E., Hernandez T.L., Barbour L.A., Friedman J.E., Krebs N.F., MacLean P.S. (2017). Early infant adipose deposition is positively associated with the *n*-6 to *n*-3 fatty acid ratio in human milk independent of maternal BMI. Int. J. Obes. (Lond.).

[43] Muley A., Muley P., Shah M. (2014). ALA, fatty fish or marine *n*-3 fatty acids for preventing DM? A systematic review and meta-analysis. Curr. Diabetes Rev..

[44] D’Andrea S., Guillou H., Jan S., Catheline D., Thibault J.N., Bouriel M., Rioux V., Legrand P. (2002). The same rat Delta6-desaturase not only acts on 18- but also on 24-carbon fatty acids in very-long-chain polyunsaturated fatty acid biosynthesis. Biochem. J..

[45] Monk J.M., Liddle D.M., Cohen D.J., Tsang D.H., Hillyer L.M., Abdelmagid S.A., Nakamura M.T., Power K.A., Ma D.W., Robinson L.E. (2016). The delta 6 desaturase knock out mouse reveals that immunomodulatory effects of essential *n*-6 and *n*-3 polyunsaturated fatty acids are both independent of and dependent upon conversion. J. Nutr. Biochem..

[46] Muhlhausler B.S., Cook-Johnson R., James M., Miljkovic D., Duthoit E., Gibson R. (2010). Opposing effects of omega-3 and omega-6 long chain polyunsaturated Fatty acids on the expression of lipogenic genes in omental and retroperitoneal adipose depots in the rat. J. Nutr. Metab..

[47] Pinel A., Pitois E., Rigaudiere J.P., Jouve C., De Saint-Vincent S., Laillet B., Montaurier C., Huertas A., Morio B., Capel F. (2016). EPA prevents fat mass expansion and metabolic disturbances in mice fed with a Western diet. J. Lipid Res..

[48] Fjaere E., Aune U.L., Roen K., Keenan A.H., Ma T., Borkowski K., Kristensen D.M., Novotny G.W., Mandrup-Poulsen T., Hudson B.D. (2014). Indomethacin Treatment Prevents High Fat Diet-induced Obesity and Insulin Resistance but Not Glucose Intolerance in C57BL/6J Mice. J. Biol. Chem..

[49] Zhao M., Chen X. (2014). Eicosapentaenoic acid promotes thermogenic and fatty acid storage capacity in mouse subcutaneous adipocytes. Biochem. Biophys. Res. Commun..

[50] Shin S., Ajuwon K.M. (2018). Lipopolysaccharide Alters Thermogenic and Inflammatory Genes in White Adipose Tissue in Mice Fed Diets with Distinct 18-Carbon Fatty-Acid Composition. Lipids.

[51] Sundaram S., Bukowski M.R., Lie W.R., Picklo M.J., Yan L. (2016). High-Fat Diets Containing Different Amounts of n3 and n6 Polyunsaturated Fatty Acids Modulate Inflammatory Cytokine Production in Mice. Lipids.

[52] Todoric J., Loffler M., Huber J., Bilban M., Reimers M., Kadl A., Zeyda M., Waldhausl W., Stulnig T.M. (2006). Adipose tissue inflammation induced by high-fat diet in obese diabetic mice is prevented by *n*-3 polyunsaturated fatty acids. Diabetologia.

[53] Blok W.L., Deslypere J.P., Demacker P.N., van der Ven-Jongekrijg J., Hectors M.P., van der Meer J.W., Katan M.B. (1997). Pro- and anti-inflammatory cytokines in healthy volunteers fed various doses of fish oil for 1 year. Eur. J. Clin. Investig..

[54] Cooper A.L., Gibbons L., Horan M.A., Little R.A., Rothwell N.J. (1993). Effect of dietary fish oil supplementation on fever and cytokine production in human volunteers. Clin. Nutr..

[55] James M.J., Gibson R.A., Cleland L.G. (2000). Dietary polyunsaturated fatty acids and inflammatory mediator production. Am. J. Clin. Nutr..

[56] Balvers M.G., Verhoeckx K.C., Meijerink J., Bijlsma S., Rubingh C.M., Wortelboer H.M., Witkamp R.F. (2012). Time-dependent effect of in vivo inflammation on eicosanoid and endocannabinoid levels in plasma, liver, ileum and adipose tissue in C57BL/6 mice fed a fish-oil diet. Int. Immunopharmacol..

[57] Spencer M., Finlin B.S., Unal R., Zhu B., Morris A.J., Shipp L.R., Lee J., Walton R.G., Adu A., Erfani R. (2013). Omega-3 fatty acids reduce adipose tissue macrophages in human subjects with insulin resistance. Diabetes.

[58] Hames K.C., Morgan-Bathke M., Harteneck D.A., Zhou L., Port J.D., Lanza I.R., Jensen M.D. (2017). Very-long-chain omega-3 fatty acid supplements and adipose tissue functions: A randomized controlled trial. Am. J. Clin. Nutr..

[59] Mendonca A.M., Cayer L.G.J., Pauls S.D., Winter T., Leng S., Taylor C.G., Zahradka P., Aukema H.M. (2018). Distinct effects of dietary ALA, EPA and DHA on rat adipose oxylipins vary by depot location and sex. Prostaglandins Leukot. Essent. Fatty Acids.

[60] Ferguson J.F., Roberts-Lee K., Borcea C., Smith H.M., Midgette Y., Shah R. (2018). Omega-3 polyunsaturated fatty acids attenuate inflammatory activation and alter differentiation in human adipocytes. J. Nutr. Biochem..

[61] Titos E., Rius B., Gonzalez-Periz A., Lopez-Vicario C., Moran-Salvador E., Martinez-Clemente M., Arroyo V., Claria J. (2011). Resolvin D1 and its precursor docosahexaenoic acid promote resolution of adipose tissue inflammation by eliciting macrophage polarization toward an M2-like phenotype. J. Immunol..

[62] Fredman G., Serhan C.N. (2011). Specialized proresolving mediator targets for RvE1 and RvD1 in peripheral blood and mechanisms of resolution. Biochem. J..

[63] Fleming I. (2014). The pharmacology of the cytochrome P450 epoxygenase/soluble epoxide hydrolase axis in the vasculature and cardiovascular disease. Pharmacol. Rev..

[64] Balvers M.G., Verhoeckx K.C., Bijlsma S., Rubingh C.M., Meijerink J., Wortelboer H.M., Witkamp R.F. (2012). Fish oil and inflammatory status alter the *n*-3 to *n*-6 balance of the endocannabinoid and oxylipin metabolomes in mouse plasma and tissues. Metabolomics.

[65] Hammond V.J., O’Donnell V.B. (2012). Esterified eicosanoids: Generation, characterization and function. Biochim. Biophys. Acta.

[66] Quehenberger O., Dahlberg-Wright S., Jiang J., Armando A.M., Dennis E.A. (2018). Quantitative determination of esterified eicosanoids and related oxygenated metabolites after base hydrolysis. J. Lipid Res..

[67] Vangaveti V.N., Jansen H., Kennedy R.L., Malabu U.H. (2016). Hydroxyoctadecadienoic acids: Oxidised derivatives of linoleic acid and their role in inflammation associated with metabolic syndrome and cancer. Eur. J. Pharmacol..

[68] Nagy L., Tontonoz P., Alvarez J.G., Chen H., Evans R.M. (1998). Oxidized LDL regulates macrophage gene expression through ligand activation of PPARgamma. Cell.

[69] White P.J., Arita M., Taguchi R., Kang J.X., Marette A. (2010). Transgenic restoration of long-chain *n*-3 fatty acids in insulin target tissues improves resolution capacity and alleviates obesity-linked inflammation and insulin resistance in high-fat-fed mice. Diabetes.

[70] Yan Y., Jiang W., Spinetti T., Tardivel A., Castillo R., Bourquin C., Guarda G., Tian Z., Tschopp J., Zhou R. (2013). Omega-3 fatty acids prevent inflammation and metabolic disorder through inhibition of NLRP3 inflammasome activation. Immunity.

[71] Rey C., Delpech J.C., Madore C., Nadjar A., Greenhalgh A.D., Amadieu C., Aubert A., Pallet V., Vaysse C., Laye S. (2019). Dietary *n*-3 long chain PUFA supplementation promotes a pro-resolving oxylipin profile in the brain. Brain Behav. Immun..

